# Does meniscal repair impact muscle strength following ACL reconstruction?

**DOI:** 10.1051/sicotj/2022016

**Published:** 2022-05-16

**Authors:** Guillaume Mesnard, Gaspard Fournier, Léopold Joseph, Jobe Gennadi Shatrov, Sébastien Lustig, Elvire Servien

**Affiliations:** 1 Orthopaedics Surgery and Sports Medicine Department, FIFA Medical Center of Excellence, Croix-Rousse Hospital, Lyon University Hospital 69004 Lyon France; 2 The University of Notre Dame, Australia, School of Medicine 6160 Sydney Australia; 3 Univ Lyon, Claude Bernard Lyon 1 University, IFSTTAR, LBMC UMR_T9406 69622 Lyon France; 4 LIBM – EA 7424, Interuniversity Laboratory of Biology of Mobility, Claude Bernard Lyon 1 University 69100 Lyon France

**Keywords:** Anterior cruciate ligament, ACL reconstruction, Meniscus, Meniscal repair, Isokinetic tests

## Abstract

*Purpose*: Meniscal lesions are commonly associated with anterior cruciate ligament (ACL) rupture. Meniscal repair, when possible, is widely accepted as the standard of care. Despite advancements in surgical and rehabilitation techniques, meniscal repair may impact muscle recovery when performed in conjunction with ACL reconstruction. The objective of this study was to explore if meniscal repairs in the context of ACL reconstruction affected muscle recovery compared to isolated ACL reconstruction. *Methods*: Fifty-nine patients with isolated ACL reconstruction were compared to 35 patients with ACL reconstruction with an associated meniscal repair. All ACL reconstructions were performed using hamstring grafts with screw-interference graft fixation. Isokinetic muscle testing was performed between six and eight months of follow-up. Muscle recovery between both groups was compared. A further subgroup analysis was performed to compare muscle recovery function of gender and meniscal tear location. Tegner scores were assessed at six months’ follow-up. *Results*: No significant differences were found between the two groups regarding muscle recovery. No difference in muscle recovery was found concerning gender. Lesion of both *menisci* significantly increased the deficit of hamstrings muscular strength at 60°/s compared to a lesion of one *meniscus* (26.7% ± 15.2 vs. 18.1% ± 13.5, *p* = 0.018) and in eccentric test (32.4% ± 26.2 vs. 18.1% ± 13.5, *p* = 0.040). No significant differences were found concerning the Tegner score. *Conclusion*: Meniscal repairs performed during an ACL reconstruction do not impact muscle recovery at 6–8 months post-operatively compared to an isolated ACL reconstruction. However, reparations of both *menisci* appear to impact hamstring muscle recovery negatively.

*Level of evidence*: III, Retrospective cohort study

## Introduction

Anterior cruciate ligament (ACL) rupture is the most frequently observed knee injury in the sporting population [1, 2], for both professional and amateur athletes [3]. Following reconstructive surgery, this patient profile commonly seeks an expeditious return to sport (RTS). Multiple factors contribute to a successful RTS, however muscle strength is an essential element, with insufficient muscle strength being associated with ACL re-rupture [4, 5], poor long-term recovery [6], and in some institutions, is itself an important criterion to authorize the return to sport. Higher post-operative quadriceps strength has been associated with a faster RTS [7].

Objectively testing muscle recovery is required to inform patient rehabilitation following ACL surgery. Isokinetic testing is one such method that provides reliable data on the targeted muscle group. Resistance is accommodated throughout a range of motion in a manner that has been demonstrated to closely resemble normal muscle function [8]. Isokinetic muscle testing of hamstring asymmetry, hamstring to quadriceps ratio, and quadriceps strength have been used following ACL reconstruction to guide rehabilitation and, in some institutions, forms are a major component of RTS criteria [9, 10].

Muscle strength following ACL reconstruction is affected by multiple factors. Meniscal lesions are one such factor and are associated with 41–55% of ACL ruptures [11–13]. *Menisci* are stabilizers, both in terms of rotation and translation [14, 15], and allow for an equal distribution of forces on the tibial articular surface [11, 16]. Preservation of meniscal lesions through surgical repair with sutures has been demonstrated to reduce the risk of arthritis development and have a protective action for the ACL graft [17, 18]. Some studies have previously observed decreased muscle strength in patients who have undergone arthroscopic surgery, including meniscectomies [19]. Post-operative rehabilitation protocols following meniscal repair may differ from an ACL reconstruction without meniscal repair, which could explain differences in the rate and degree of muscle recovery.

The objective of this study was to explore if meniscal repair, in the context of ACL reconstruction, is associated with inferior muscle recovery compared to isolated ACL reconstruction.

## Materials and methods

### Participants (Figure 1)

A retrospective study was performed based on prospectively collected data. Inclusion criteria were patients who had undergone a primary ACL reconstruction in one center (Orthopaedic Surgery Department, Hopital Croix Rousse) between August 2018 and October 2020. ACL rupture and meniscus lesions were assessed by clinical examination and MRI. There were a total of 302 patients identified in this study period. Exclusion *criteria* were bone patella bone graft reconstruction, quadriceps tendon reconstruction, lack of isokinetic test at a minimum of six months post-operatively, previous surgery to either knee for any reason, non-repairable meniscus, multi-ligament knee injury, and associated anterolateral ligament reconstruction.

**Figure 1:**
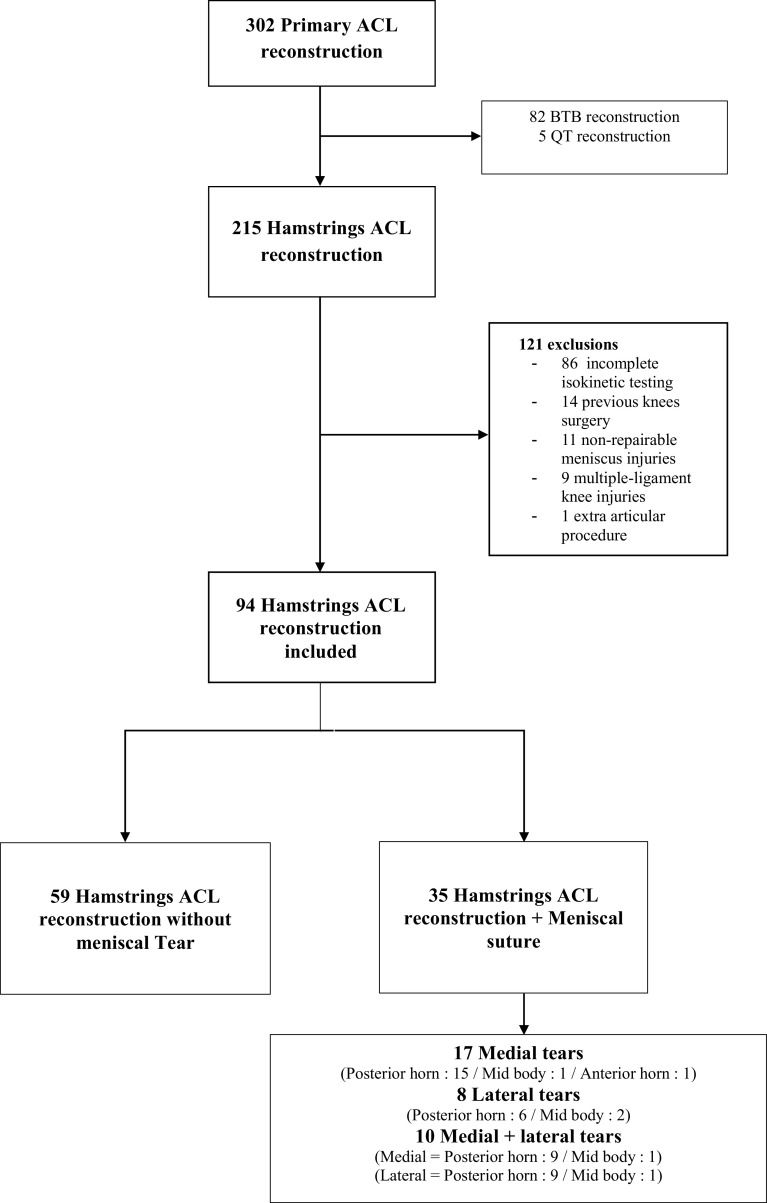
Flow chart.

Reparability of the meniscus was assessed intraoperatively: meniscal flap and lesion without healing potential (white/white lesions or degenerative meniscus) were the rare indications of meniscectomy.

A total of 94 patients were eligible and included in two groups: ACL reconstruction without meniscal repair (ACLR) (*n* = 59) and ACL reconstruction with meniscal repair (ACLR + MR) (*n* = 35).

The location of meniscal lesions is described in the flow chart (Figure 1). Baseline characteristics of the population are described in Table 1. There was no significant difference between groups for age, sex, BMI, range of motion, time from injury to surgery, and pre-operative Tegner scores.

**Table 1:** Preoperative characteristics.

	Hamstrings ACLR (*n* = 59)			Hamstrings ACLR + Meniscal repair (*n* = 35)			
	*N* (%)	Mean ± SD	(Min–Max)	*N* (%)	Mean ± SD	(Min–Max)	*p*-value*
Characteristics							
Female sex	37 (62.7%)			16 (45.7%)			n.s.
Age (yrs)		30.1 ± 9.8	(15.0–64.0)		31.7 ± 10.5	(14.0–51.0)	n.s.
BMI		23.7 ± 3.3	(18.4–32.2)		23.4 ± 2.8	(18.7–29.0)	n.s.
Tegner score		5.2 ± 1.8	(2.0–10.0)		5.1 ± 2	(1.0–9.0)	n.s.
Time from injury to surgery (months)		8.1 ± 21.6	(1.3–168.0)		11.4 ± 24	(0.5–120.0)	n.s.
Preoperative examination							
Range of motion							
Recurvatum	21 (35.5%)			8 (22.9%)			n.s.
Fixed flexion deformity	5 (8.5%)			7 (20.0%)			n.s.
Flexion (°)		132.0 ± 14.4	(80.0–150.0)		128.7 ± 14.9	(90.0–145.0)	n.s.
Lachman-Trillat							n.s.
Grade 1 (soil endpoint)	53 (89.8%)			33 (100.0%)			
Grade 2 (delayed firm endpoint)	6 (10.2%)			2 (0.0%)			
Grade 3 (firm endpoint)	0 (0.0%)			0 (0.0%)			

SD: standard deviation, ACLR: anterior cruciate ligament reconstruction.

* Exact Fischer test and Student *t*-test, n.s.: not significant

### Surgery

Patients were managed, operated and reviewed routinely according to standard institutional practices for ACL injuries in our institution.

All patients underwent an ACL reconstruction using a hamstrings graft. The surgical procedure was performed with a thigh tourniquet. A vertical two-centimeter incision was made over the pes anserinus. Semitendinosus and gracilis tendons were harvested with a closed tendon stripper and prepared in a four-strands-single-bundle graft. The graft was measured to choose the drilling diameter. If present, meniscal lesions were first assessed for suitability for repair, and if deemed appropriate, meniscal repairs were performed as follows: posterior horn repairs were performed using an all-inside method (FastFix, Smith & Nephew, London, UK), one anterior lesion was repaired using an outside-in technique using non-resorbable sutures (PDS 2-0) [20]. Repairs of bucket-handle lesions were performed by combining all-inside techniques (FastFix, Smith & Nephew, London, UK) and inside-out techniques. One patient had a root lesion which was repaired using an all-inside suture technique.

Meniscal repairs were performed in the stressed valgus for the medial meniscus and Cabot’s position for the lateral. Following repair, an outside-in femoral tunnel was drilled followed by an outside-in tibial tunnel. The graft was inserted into the femoral tunnel under arthroscopic control. Graft fixation in cases was with interference screw for both tunnels (Biosure^®^, Smith & Nephew, Memphis, TN). The femoral part of the graft was fixed first, followed by tibial fixation at 30° of flexion after cycling the knee. At the completion of surgery, a knee brace was applied and unlocked to allow flexion to 90° for all patients.

### Rehabilitation

All patients received a standardized post-operative rehabilitation program which commenced the day after the surgery. Immediate full weight-bearing was allowed. For all patients, the knee brace was used until sufficient quadriceps control was achieved as determined by a physiotherapist. Full range of motion was allowed for the ACLR group. ACLR + MR group was limited to 90° of flexion for the first six weeks, after which full movement was allowed.

Physiotherapy consisted of the closed kinetic chain and hamstring eccentric exercises, which were performed at home or in a rehabilitation center.

Cycling was allowed between six weeks and three months, and pivot sports could only be practiced if isokinetic testing after six months indicated a successful rehabilitation.

### Follow-up

All patients received a standardized follow-up protocol consisting of assessment at three weeks by a sports medicine physician and then at six weeks by the orthopedic surgeon. Isokinetic testing was performed between six and eight months post-operatively by a sports medicine physician. Tegner scores [21] were collected pre-operatively and at six months post-operatively through online questionnaires.

### Muscle strength testing

Isokinetic muscle testing was performed using the Con-trex^®^ (Physiomed Elektromedizin AG, Germany) machine. Patients underwent a standardized protocol consisting of a 15-min warm-up on a stationary bike, beginning with the non-operated side for each testing step.

Before each sequence, submaximal trials were done for familiarization and training.

Tests started with the concentric step: three repetitions were performed at 60°/s, then three at 240°/s for each knee. The range of motion was 70° (20–90°). Then, eccentric tests were performed with three maximal repetitions at 30°/s. Peak torque, in Newton per meter (Nm) and Nm/kg, percentage deficits of the affected side compared to the healthy side were collected for each muscle group. The hamstrings/quadriceps ratio was calculated for each speed.

### Ethics

All procedures performed in studies involving human participants were in accordance with the ethical standards of the institutional and/or national research committee and with the 1964 Helsinki declaration and its later amendments or comparable ethical standards.

### Statistical analysis

Statistical analyses were performed using the XLSTAT software (V2021.1, Addinsoft, Paris, France). Categorical outcomes were compared using the chi-squared test. Quantitative variables were compared using the Student *t*-test. For all analyses, a *p*-value < 0.05 was considered statistically significant.

## Results

### Isokinetic tests

Results of the isokinetic tests are presented in Table 2. Strength was similar between the two groups for the concentric and eccentric tests. No significant difference was found between groups in strength deficit for all the tests.

**Table 2:** Isokinetics results.

	Hamstrings ACLR *(n* = 59)		Hamstrings ACLR + Meniscal repair (*n* = 35)		
	Mean ± SD	(Min–Max)	Mean ± SD	(Min–Max)	*p*-value*
Concentrics tests 240°/s					
Q strength (Nm)	72.5 ± 33.0	(19.1–152.0)	78.1 ± 32.0	(20.6–155.7)	n.s.
Q deficit (%)	23.3 ± 18.3	(−7.4–69.4)	21.8 ± 21.1	(−45.8–71.3)	n.s.
Q torque (Nm)/kg	1.0 ± 0.4	(0.2–1.9)	1.0 ± 0.3	(0.3–1.8)	n.s.
H strength (Nm)	57.6 ± 22.2	(21.0–121.1)	61.3 ± 24.1	(20.1–114.7)	n.s.
H deficit (%)	13.9 ± 12.2	(−14.3–43.4)	14.2 ± 19.8	(−39.7–60.3)	n.s.
H torque (Nm)/kg	0.8 ± 0.3	(0.2–2.0)	0.8 ± 0.3	(0.3–1.5)	n.s.
Concentrics tests 60°/s					
Q strength (Nm)	105.4 ± 49.7	(21.9–234.1)	116.9 ± 51.5	(26.7–217.0)	n.s.
Q deficit (%)	24.3 ± 21.2	(−28.7–75.9)	21.8 ± 24.8	(−41.5–76.5)	n.s.
Q torque (Nm)/kg	1.5 ± 0.5	(0.4–2.8)	1.6 ± 0.6	(0.5–2.9)	n.s.
H strength (Nm)	73.3 ± 25.5	(31.0–132.0)	78.2 ± 32.7	(25.8–143.0)	n.s.
H deficit (%)	13.3 ± 13.9	(−26.2–46.1)	15.4 ± 18.1	(−28.0–60.0)	n.s.
H torque (Nm)/kg	1.0 ± 0.3	(0.5–1.9)	1.0 ± 0.4	(0.0–1.9)	n.s.
Eccentrics tests 30°/s					
H strength(Nm)	88.3 ± 29.6	(28.3–157.0)	96.2 ± 37.8	(17.1–178.0)	n.s.
H deficit (%)	24.5 ± 13.6	(−14.6–50.0)	22.2 ± 18.7	(−19.0–75.1)	n.s.
H torque (Nm)/kg	1.3 ± 0.4	(0.6–2.5)	1.4 ± 0.4	(0.7–2.5)	n.s.
Ratio (H/Q)					
Ratio 240 (normal 0.7)	0.86 ± 0.31	(0.49–1.90)	0.83 ± 0.26	(0.42–1.77)	n.s.
Ratio 60 (normal 0.6)	0.81 ± 0.41	(0.37–2.74)	0.71 ± 0.24	(0.36–1.70)	n.s.
Ecc ratio (normal 1)	1.38 ± 0.64	(0.40–3.67)	1.28 ± 0.34	(0.73–2.05)	n.s.

H: hamstrings; Q: quadriceps; Nm: Newton·meter; kg: kilogram; Strength(Nm): maximal strength operated knee; Ecc ratio: eccentric ratio H30/Q240; SD: standard deviation.

* Student *t*-test, n.s.: not significant.

### Ratio (hamstrings/quadriceps)

No significant difference was found concerning the hamstring/quadriceps ratio.

### Subgroups analysis: function of gender

No significant difference concerning muscle recovery was found when comparing genders.

### Subgroups analysis: function of tear location

A subgroup analysis was made as a function of the location of meniscal tears. No significant difference was found between isolated lateral or isolated medial *meniscus* tears. However, when both *menisci* (medial and lateral) had a tear, muscle recovery was significantly worse, and muscular strength deficit was globally higher compared to the subgroup composed with only one *meniscus* tear. Significance was found for the hamstrings, in the concentric test, at 60°/s and eccentric test. (Table 3)

**Table 3:** Isokinetics results according to meniscal lesion.

	Medial or lateral meniscal lesion (*n* = 25)		Medial + Lateral meniscal lesions (*n* = 10)		
	Mean ± SD	(Min–Max)	Mean ± SD	(Min–Max)	*p*-value*
Concentrics tests 240°/s					
Q deficit (%)	19.9 ± 16.5	(−20.0–48.6)	26.6 ± 30.1	(−45.8–71.3)	n.s.
H deficit (%)	10.7 ± 20.5	(−39.8–60.3)	23.0 ± 15.2	(−3.1–45.5)	n.s.
Concentrics tests 60°/s					
Q deficit (%)	17.8 ± 21.9	(−41.5–60.8)	31.8 ± 29.8	(−27.8–76.5)	n.s.
H deficit (%)	10.9 ± 17.4	(−28.3–60.0)	26.7 ± 15.2	(1.8–45.1)	**0.018**
Eccentrics tests 30°/s					
H deficit (%)	18.1 ± 13.5	(0.7–53.6)	32.4 ± 26.2	(−19.0–75.1)	**0.040**
Ratio (H/Q)					
Ratio 240 (normal 0.7)	0.79 ± 0.23	(0.43–1.49)	0.92 ± 0.34	(0.66–1.77)	n.s.
Ratio 60 (normal 0.6)	0.68 ± 0.18	(0.35–1.21)	0.78 ± 0.34	(0.57–1.70)	n.s.
Ecc ratio (normal 1)	1.28 ± 0.34	(0.73–1.80)	1.28 ± 0.37	(0.83–2.05)	n.s.

H: hamstrings; Q: quadriceps; Ecc ratio: eccentric ratio H30/Q240; SD: standard deviation.

Bold values indicate at *p* < 0.05.

* Student *t*-test, n.s: not significant.

### Operative and tourniquet time

Operative and tourniquet time was slightly longer in the group with both medial and lateral tears compared to the isolated tear group; however, differences were not statistically significant. For patients with tears in both the lateral and medial *menisci* versus the group with either isolated lateral or medial lesions, the mean tourniquet time was 71.3 ± 17.3 versus 61.4 ± 13 min (*p* = 0.072). Similarly, for operative time, no significant difference was found between the groups (80.6 ± 19.8 vs. 70.3 ± 13, *p* = 0.077).

### Tegner score

The mean Tegner score at six months post-operative for the isolated ACLR group was 5.0 ± 1.9 versus 4.5 ± 2.1 in the ACLR associated with the meniscal repair group. No significant difference was found (*p* = 0.262).

## Discussion

The most important finding of this study was that unilateral meniscal repair performed during ACL reconstruction does not affect the muscle strength of quadriceps and hamstrings at 6–8 months post-surgery. When tears were repaired in both compartments, hamstrings muscle strength was statistically reduced.

Regaining muscle strength is an integral part of a successful rehabilitation following ACLR. Meniscal lesions are commonly associated with ACL rupture, occurring in 41–55% of cases [11–13]. Orthopaedic surgeons have for many years understood the importance of meniscal preservation and the value of a conservative approach when treating tears [22, 23]. Successful meniscal repair results in improvements in subjective knee scores, is protective against the development of osteoarthritis and improves the stability of the knee [24–27]. However, meniscal repair often limits rehabilitation, with restrictions on flexion range or weight-bearing allowed [28].

Previous studies have found a negative association between isolated meniscal repair and muscle strength. Stensrud et al. [29] demonstrated that the muscular strength of limbs with meniscal injuries was significantly weaker. Eitzen et al. [30] assessed similar results, finding less muscular strength for patients suffering from degenerative meniscus tears. McLeod et al. [19] objectified a decreased muscular strength for patients who underwent arthroscopy for meniscectomy. Meniscal repair with concurrent ACLR may limit the post-operative muscular rehabilitation, and this could slow recovery.

The results of this study reject our hypothesis that ACLR, in combination with meniscal repairs, has worse muscle strength in hamstring and quadriceps muscle groups, and our findings are in line with previous literature. Lepley et al. [31] found no differences in quadriceps strength and activation at the time of return to sport in 46 patients split into three groups: ACLR without meniscal surgery, ACLR with meniscectomy, and ACLR with meniscal repair. However, strength was only assessed at a time when patients had been deemed suitable to return to sport, which itself requires satisfactory muscle recovery and strength, potentially confounding the results. Hall et al. [32] studied the isokinetic max extensor knee strength and the knee adduction moment at 12 and 24 months post-ACLR, with no difference in muscle strength or gait being demonstrated if a meniscal procedure was performed. Wenning et al. [33] examined the peak flexion and extension torque after ACLR six months post operatively, finding no significant difference between patients operated for ACLR and meniscal surgery compared to patients who received isolated ACLR. This study included multiple graft types and surgeons, with variable rehabilitation protocols, according to the type of meniscal lesion. In the present study, the range of motion was restricted from 0° to 90° flexion for six weeks post operatively, and full weight-bearing was allowed. Despite these differences in rehabilitation protocols, no strength deficit was identified. Similarly, Wenning et al. [33] limited rehabilitation, restricting the flexion but also limited weight-bearing, without a deleterious effect on muscle recovery.

In the current study, subgroup analysis did not show differences in strength recovery based on the gender of patients. However, when examining the impact of the location of the meniscal injury, patients who received both medial and lateral *meniscus* repairs demonstrated a greater muscular deficit, which was more pronounced in the hamstring group than the quadriceps. Differences between patient groups were greatest at 60°/s for eccentric testing. When one *meniscus* was repaired (medial or lateral), no difference was found between medial or lateral compartments. This is in contrast to Wenning et al. [33], who also examined the impact of the location of the meniscal lesion, finding larger muscular strength deficits with lateral *meniscus* repairs compared to both *menisci* and medial *meniscus* tear. However, these differences did not reach statistical significance.

The etiology of the differences observed between groups based on tear location in this study is not clear. The accentuated deficit when two lesions of the *menisci* are repaired may be due to greater pain after surgery, which could slow rehabilitation and has been shown previously to have a negative effect on muscle activation [34, 35].

Another possibility is the influence of tourniquet time. Double meniscal repairs raise the complexity and length of surgery, with a natural increase in the duration of tourniquet time. However, while plausible, the effect of the tourniquet use on muscular strength remains unclear [36, 37].

The findings in the present study have implications for patients following ACLR requiring meniscal repair in both tibiofemoral compartments. The orthopedic surgeon, sports clinician, and physiotherapist should tailor rehabilitation to focus more intensively on muscle strengthening in these patients, with particular attention given to hamstring strength. Failure to do so may slow a return to sport, lead to increased laxity, and could contribute to graft failure. However, patients can be reassured that unilateral meniscal repair does not delay muscle strength recovery at six months following ACLR.

One of the strengths of this study is the uniformity of graft choice for all ACLRs. All patients had a four strand graft using *gracillis* and *semitendinosus* tendons. Graft type for ACLR has been shown to affect the course of post-operative muscle recovery [38–40]. Also, all the patients were followed by a single team of sports medicine physicians, who performed the isokinetic tests with one protocol, and in conjunction with our physiotherapists, oversaw the rehabilitation of all patients in this study.

Our study has several limitations. The number of patients included in this study is small, particularly in the meniscal repair group. While this does limit the strength of our findings, they are consistent with published data, and the size of our cohort is comparable to previous studies. Our cohort did not include a meniscectomy group, but the addition of such a group could precise the exact effect of meniscal repair. Finally, we analyzed the muscular strength at a precise time (six months follow-up). Pre-operative isokinetic muscle testing would control baseline muscle function and explore the evolution of the muscle recovery and the delay in acquiring muscular strength symmetry.

## Conclusion

This study revealed that meniscal repairs performed during an ACL reconstruction do not impact muscle recovery at 6–8 months post-operatively compared to an isolated ACL reconstruction. However, reparations of both *menisci* impact negatively the hamstrings muscle recovery. These findings have implications for the rehabilitation of patients undergoing ACLR with associated meniscal repairs in both the medial and lateral compartments.
